# Adenoviral vaccine induction of CD8^+^ T cell memory inflation: Impact of co-infection and infection order

**DOI:** 10.1371/journal.ppat.1006782

**Published:** 2017-12-27

**Authors:** Lian N. Lee, Beatrice Bolinger, Zoltan Banki, Catherine de Lara, Andrew J. Highton, Julia M. Colston, Claire Hutchings, Paul Klenerman

**Affiliations:** 1 Peter Medawar Building and Translational Gastroenterology Unit, Oxford, United Kingdom; 2 Schweizerischer Apothekerverband, pharmaSuisse, Bern, Switzerland; 3 Division of Virology, Innsbruck Medical University, Innsbruck, Austria; Emory Vaccine Center, UNITED STATES

## Abstract

The efficacies of many new T cell vaccines rely on generating large populations of long-lived pathogen-specific effector memory CD8 T cells. However, it is now increasingly recognized that prior infection history impacts on the host immune response. Additionally, the order in which these infections are acquired could have a major effect. Exploiting the ability to generate large sustained effector memory (i.e. inflationary) T cell populations from murine cytomegalovirus (MCMV) and human Adenovirus-subtype (AdHu5) 5-beta-galactosidase (Ad-lacZ) vector, the impact of new infections on pre-existing memory and the capacity of the host’s memory compartment to accommodate multiple inflationary populations from unrelated pathogens was investigated in a murine model. Simultaneous and sequential infections, first with MCMV followed by Ad-lacZ, generated inflationary populations towards both viruses with similar kinetics and magnitude to mono-infected groups. However, in Ad-lacZ immune mice, subsequent acute MCMV infection led to a rapid decline of the pre-existing Ad-LacZ-specific inflating population, associated with bystander activation of Fas-dependent apoptotic pathways. However, responses were maintained long-term and boosting with Ad-lacZ led to rapid re-expansion of the inflating population. These data indicate firstly that multiple specificities of inflating memory cells can be acquired at different times and stably co-exist. Some acute infections may also deplete pre-existing memory populations, thus revealing the importance of the order of infection acquisition. Importantly, immunization with an AdHu5 vector did not alter the size of the pre-existing memory. These phenomena are relevant to the development of adenoviral vectors as novel vaccination strategies for diverse infections and cancers. (241 words)

## Author summary

Many natural pathogens elicit large antigen-specific long-lived effector memory CD8 T cell responses that contribute to pathogen protection and control. This strategy is also employed by novel CD8 T cell vaccine vectors. In the real world, the host is likely to encounter a number of such pathogens at different times but the effect of pre-existing effector memory pools on the development of new CD8 T cell responses against unrelated infections, and vice versa, is still unclear. We address this through mouse models of sequential infection with MCMV and an Ad-Hu5 vaccine vector. We find that the host is able to accommodate and accumulate multiple new specificities of inflating effector memory populations at different times but certain infections, such as acute MCMV, appear to rapidly deplete existing memory populations via a Fas-dependent pathway. Additionally, immunization with vectors based on human adenovirus subtype 5 do not appear to detrimentally affect the host’s pre-existing effector memory pool. (154 words)

## Introduction

Immunologic memory is critical for host defense against pathogens and tumours and underpins the design of modern vaccines. Indeed, many current T cell vaccination strategies against pathogens or tumours aim to elicit long-term CD8 T cell effector memory responses [1–9]. However, the factors that govern the long-term maintenance of T cell memory, particularly effector memory, have not been fully elucidated. It is also unclear as to whether there is an upper limit to the capacity of the memory compartment to accommodate multiple epitopes or if new epitopes are generated and expand at the expense of pre-existing T cell memory populations. There is evidence supporting both assertions; studies indicate that pre-existing memory is eroded after infection with a subset of pathogens[10–12]. By contrast others [13–15] show that the T cell compartment is able to accommodate ever-increasing numbers of specificities. What is clear and is supported by more recent data, is that the infection history of the host can influence the response to new, unrelated pathogens and vaccines.[16]

Inflating memory responses represent a subset of long-lived epitope-specific CD8 T cell memory responses which were initially identified in a number of natural persistent murine and human infections[17–19] and recently in a murine model of infection with a non-replicative human adenovirus type 5 construct expressing the β-galactosidase gene [20–22]. These cells maintain a functional effector-memory phenotype, accumulate at high-frequencies in multiple compartments [23–25] and are able to provide rapid front-line protection against infection [26–28]. Pertinently, our group has shown that these inflating cells share a conserved phenotype with effector CD8 T cells raised upon immunization with a replication-deficient Chimpanzee Adenovirus subtype 3 vaccine vector encoding a Hepatitis C antigen [22].

Inflating epitopes may therefore serve as useful tools to measure effects of perturbations in the size of individual T cell epitope populations under different conditions. Two models of memory inflation have been developed in mice; the first, infection with murine cytomegalovirus (MCMV), a natural mouse pathogen, gives rise to multiple populations of antigen-specific inflating T cell populations as well as conventional memory T cells and recapitulates many features of cytomegalovirus (CMV) infection in humans [24,28]. The second model uses a non-replicating Adenovirus containing the beta-galactosidase gene (Ad-lacZ), and when delivered into C57BL/6 mice gives rise to an inflating population (βgal_96-103,_ D8V) and conventional CD8 T cell population (βgal_497-504,_ I8V) against beta-galactosidase [20].

By tracking these inflating populations in various co-and-sequential infection scenarios, we were able to determine the long-term fate of these populations in the face of subsequent infections with unrelated pathogens and also whether the presence of large pre-existing inflating T cell populations affect the generation of new inflating T cell epitopes.

Our findings indicate that the T cell memory compartment is in principle able to accommodate multiple inflating and central memory T cell pools from unrelated pathogens. Furthermore, multiple inflation and central memory responses against epitopes against two unrelated pathogens are able to develop simultaneously. We found that AdHu5-induced inflating responses could develop without impacting the pre-existing inflation population. In contrast, acute MCMV infection results in rapid depletion of pre-existing Ad-lacZ-induced inflating memory T cells. Nonetheless, the depleted inflating population could be successfully reestablished in the blood and organs by boosting with the Ad-lacZ vector, indicating that the restriction on the number of inflationary responses does not lie at the level of ‘immunological space’. Attrition of the existing memory T cell compartment is likely a specific by-product of infection by the pathogen and may have an important impact in shaping immunologic memory in certain settings after CMV infection and possibly related infections. Thus the order in which pathogens are encountered during the lifetime of the host can have a profound impact on long-term CD8 T cell effector memory immune responses.

## Results

### Multiple inflating responses from individual or two unrelated pathogens may develop simultaneously

**Fig 1:**
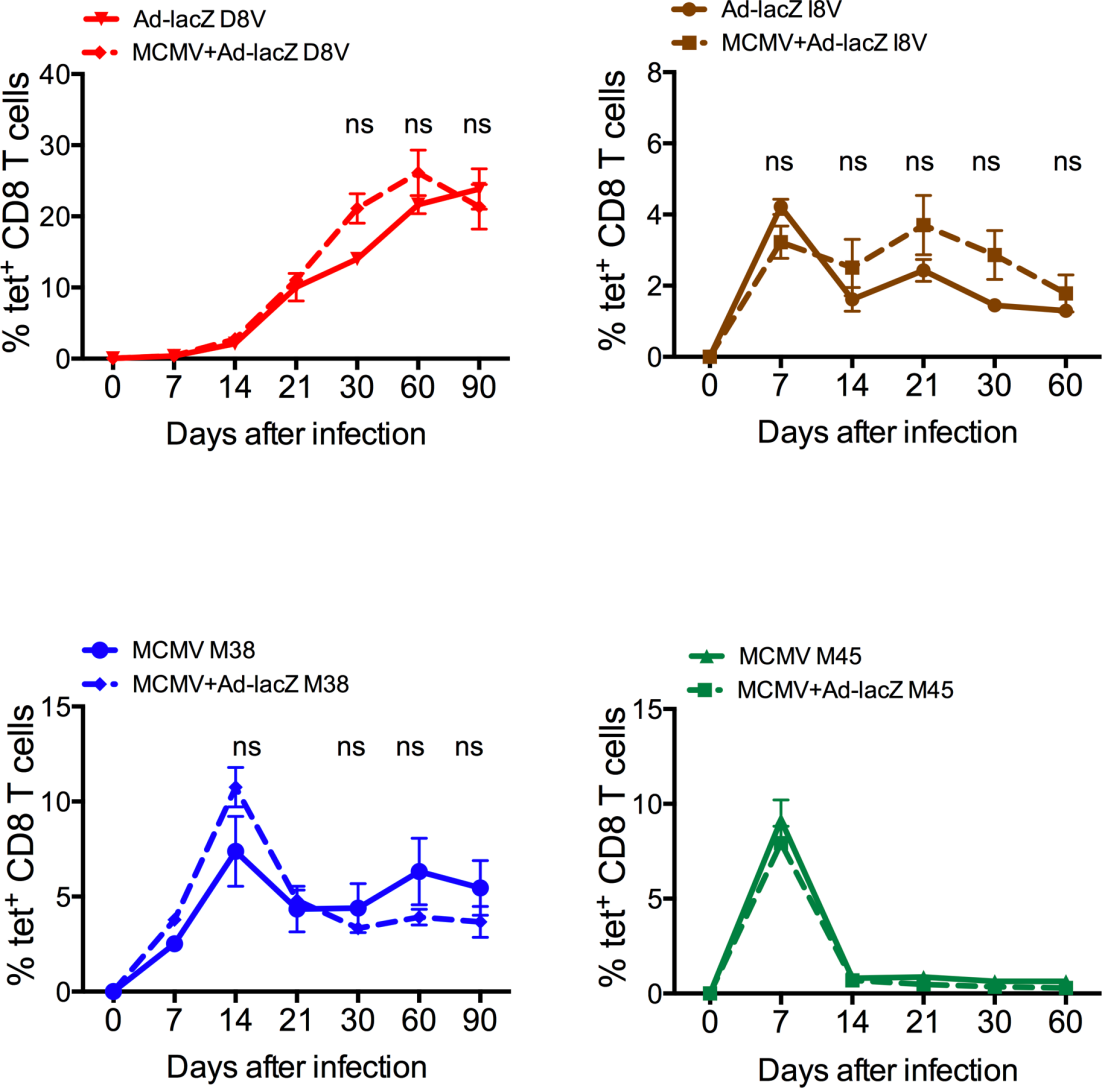
Simultaneous infection with MCMV and Ad-lacZ. Groups of C57BL/6 mice were co-infected with a mixture of 2x10^9^ pfu Ad-LacZ and 5x10^6^ pfu MCMV by the i.v. route (N = 4). Groups of mice were infected with only MCMV or Ad-lacZ as control (N = 3). At the indicated time-points post-infection blood was sampled and the levels of Ad-lacZ-specific inflating memory (D8V, upper left) and central memory (I8V, upper right) and MCMV-specific inflating memory (M38, lower left) and central memory (M45, lower right) were measured by *ex vivo* tetramer staining.

To determine whether the immune system is able to accommodate multiple inflationary responses to epitopes from different pathogens, mice were infected with MCMV and Ad-lacZ simultaneously by the intravenous (i.v.) route. The levels of inflating and central memory epitopes for the respective viruses were measured by tetramer staining of blood lymphocytes. As shown in Fig 1, co-infection does not appear to reduce the size of the inflating and central memory epitopes-specific responses compared to single infections. Additionally, multiple inflating memory populations may develop from a single virus. As shown in S1A and S1B Fig, C57BL/6 and BALB/c hybrid mice (F1) were able to generate inflating responses specific to both parental strains. Therefore, the host appears able to support the development of multiple inflating and central memory responses against two unrelated infections at the same time.

### Existing inflated CD8^+^ T cell responses are not affected by infection with Ad-lacZ and the development of a new inflating response

**Fig 2:**
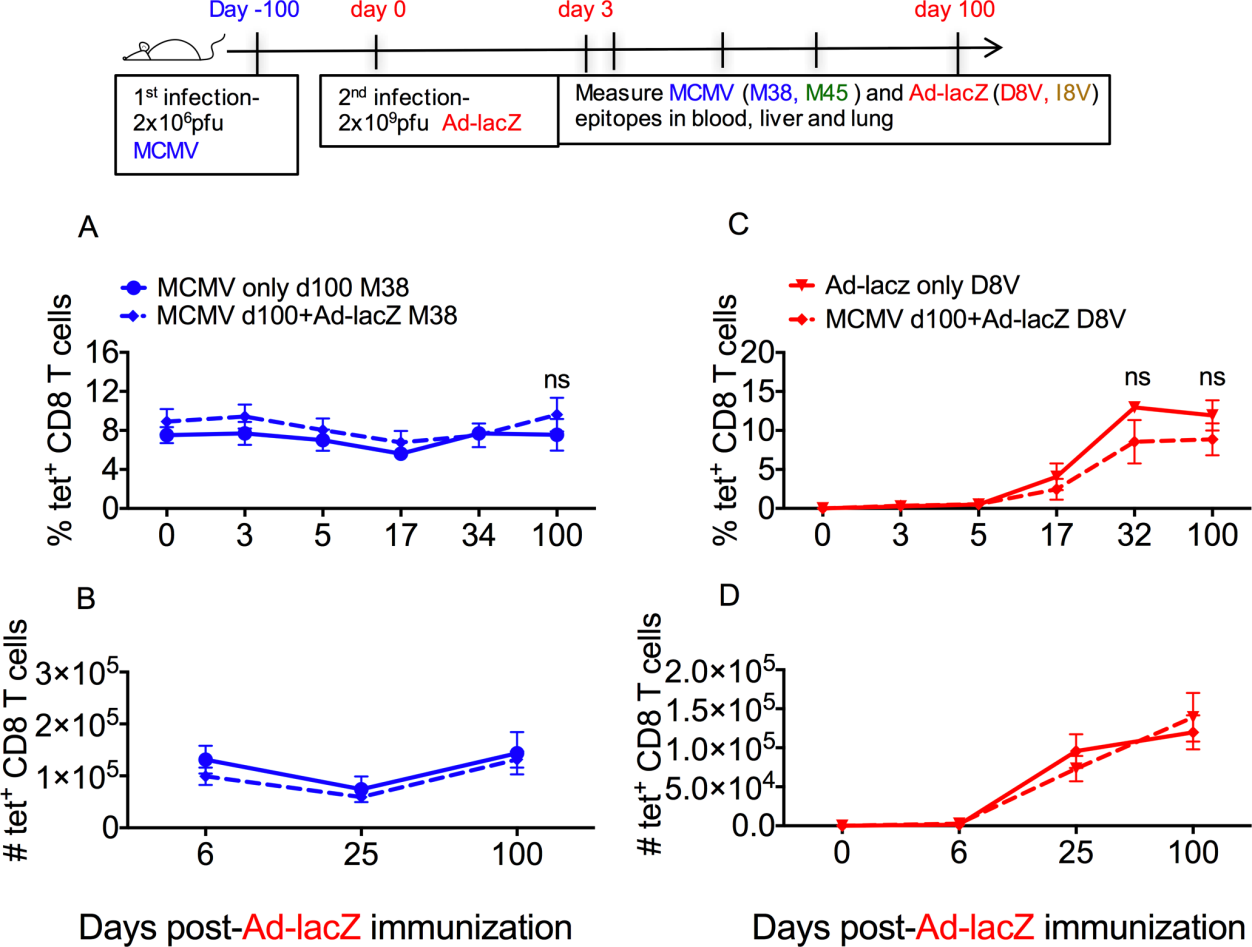
Sequential infection with MCMV followed by Ad-LacZ. (A) C57BL/6 mice were first infected with MCMV, then >50 days later were infected with Ad-lacZ. The size of the pre-existing MCMV specific M38 effector memory response after Ad-lacZ infection was measured in the blood and liver by *ex-vivo* tetramer staining. The percentage of pre-existing M38 response in the blood and (B) the absolute numbers in the liver after immunization with Ad-lacZ vector was determined at the indicated time points. (C) The kinetics and magnitude of the new inflating response (D8V) in groups of mice with latent MCMV was also measured by *ex vivo* tetramer staining compared against uninfected mice. The percentage of D8V tetramer+ cells in the blood and (D) the absolute numbers in the liver was measured at the indicated time points. The figures show the mean from 3–8 mice per time point obtained from 2 independent experiments. p values were measured by Mann-Whitney tests. *p<0.05.

The results of the first experiments indicated that mice were able to simultaneously support multiple large, inflated responses originating from infection with multiple or single viruses. In order to determine if acquiring new infections may impact upon pre-existing memory populations, the established inflating populations of Ad-lacZ and MCMV-specific cells were employed as markers. Mice were first infected with MCMV, the infection was allowed to progress to the chronic phase and then they were injected i.v. with Ad-lacZ. Infection of MCMV and Ad-lacZ by the i.v. route typically generates large populations of effector T cells in circulation as well as in the liver and lungs [20], thus the effect of Ad-lacZ on the development of the inflating D8V response was followed in these organs. The impact of the new inflating population on the pre-existing MCMV population was investigated by tracking the M38 response early after Ad-lacZ infection and up to 100 days afterwards. We found that subsequent infection with Ad-lacZ did not alter the size of the MCMV M38-specific population (Fig 2A and 2B, S2A–S2C Fig). A similar observation was made in the pre-existing MCMV-specific conventional memory response to epitope M45, where levels were unaltered after Ad-lacZ infection (S2D–S2F Fig). The level of M38-specific T cells in circulation and in non-lymphoid organs remained similar to the size of MCMV-only mice and the population remained stable for extended periods. Therefore, the presence of the new developing population of inflated cells did not impact upon the pre-existing population of effector T cells.

Similarly, despite the presence of existing populations of inflated cells, these did not limit the size of the newly developing Ad-lacZ inflating population (Fig 2C and 2D and S2G and S2H Fig) or the conventional memory population (S2I–S2K Fig). Thus the host is able to support multiple populations of epitope-specific T cells which may develop at different periods of time without any evident cost to the size of the existing or the newly generated effector memory population.

### The order in which the host acquires infections may affect the size of the pre-existing inflating/effector memory compartment

**Fig 3:**
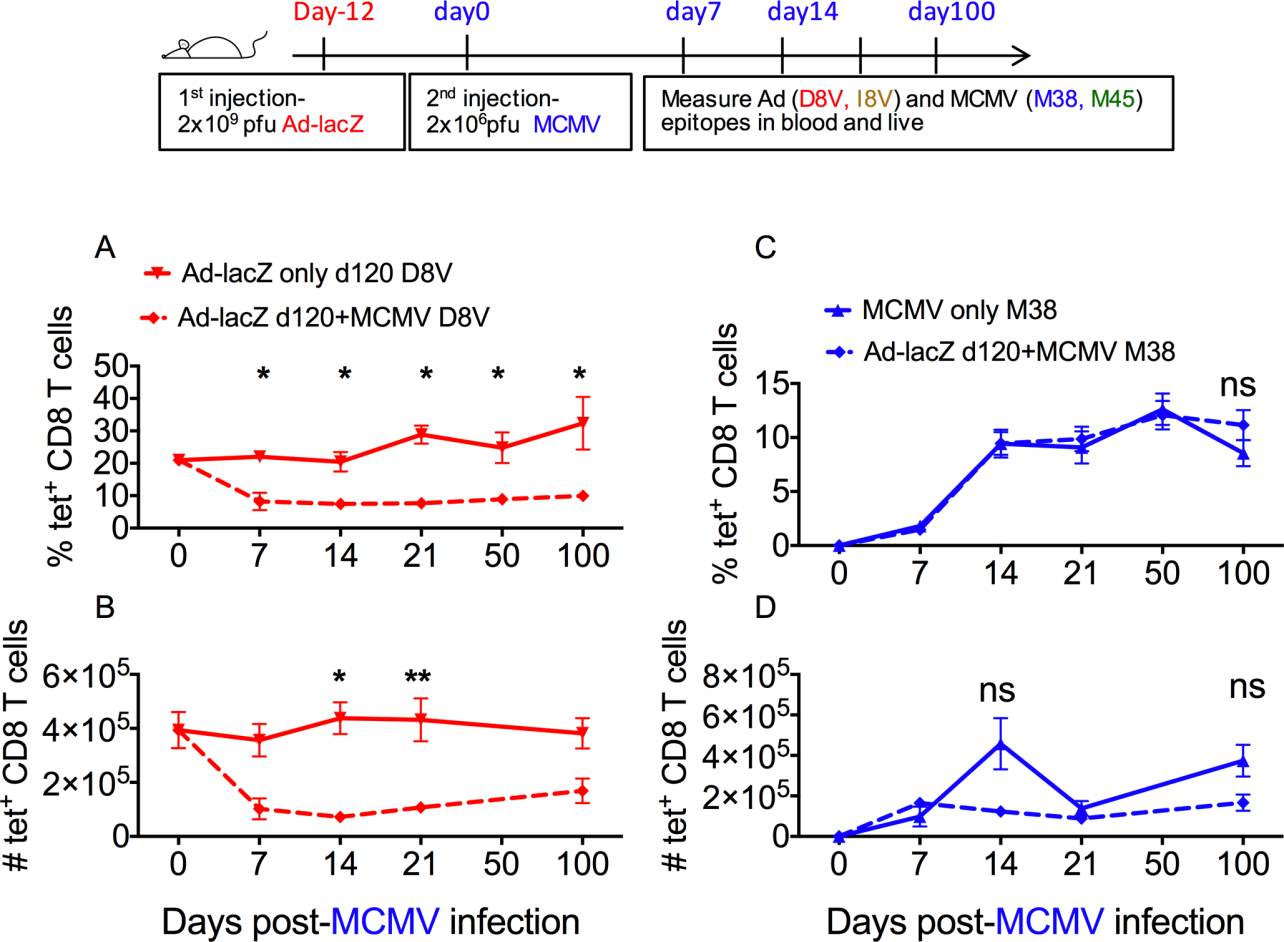
Sequential infection with Ad-LacZ followed by MCMV. C57BL/6 mice were first immunized with Ad-lacZ, then >50 days later were infected with MCMV. The size of the pre-existing Ad-lacZ specific M38 effector memory response after MCMV infection was measured in the blood and liver by ex-vivo tetramer staining. (A) The percentage of pre-existing inflating D8V tetramer+ population in the blood and (B) the absolute numbers in the liver after MCMV infection was determined at the indicated time points. (C) The kinetics and magnitude of the new inflating response (M38) in groups of mice previously immunized with Ad-lacZ was also measured by *ex vivo* tetramer staining and compared against uninfected mice. The percentage of M38 tetramer^+^ cells in the blood and (D) the absolute numbers in the liver was measured at the indicated time points. The figures show the mean from 3–8 mice per time point obtained from 2 independent experiments. p values were measured by Mann-Whitney tests followed by Dunn’s multiple comparison. *p<0.05, **p<0.005.

Next we reversed the order of infection, whereby mice initially infected with Ad-lacZ were then infected with MCMV (Fig 3). The impact of MCMV infection on the D8V population in the circulation and in non-lymphoid tissue was measured. In contrast to what was observed previously, the levels of D8V–specific T cells dropped markedly after infection with MCMV (Fig 3A and 3B and S3A and S3B Fig). This reduction was most pronounced in the blood and liver, where the populations were reduced by approximately 50% compared to non-infected Ad-lacZ immune mice, but also occurred to a lesser extent in the lungs (S3C Fig). The depletion of the existing D8V population was sustained for a long period, with the population not recovering in the blood, although there were indications of slow recovery of the numbers in the liver and lung by day 100 post-MCMV (Fig 3B and S3C Fig). The pre-existing response to Ad-lacZ central memory epitope I8V was also followed and while it exhibits a trend towards reduction after acute MCMV infection, the fall was not as steep and there was large variation within the groups (S3D–S3F Fig).

As in the previous experiment, the development of the new inflating population (MCMV-specific M38 epitope) was not altered by the presence of Ad-lacZ memory cells in the blood, liver (Fig 3C and 3D) or lungs (S3G and S3H Fig) and this was also observed in the new developing central memory population (S2I–S2K Fig). Where mice were first infected with MCMV, then infected with Ad-lacZ and subsequently re-infected i.v. with another dose of MCMV, no early alteration in the size of the D8V population occurred, confirming that events in primary MCMV infection were responsible for attrition (S4A Fig). Attrition of pre-existing D8V tetramer+ cells was also observed after infection with lower doses of MCMV, albeit at a smaller magnitude and with delayed kinetics (S4B Fig). Therefore, unlike an Ad-lacZ infection, a primary MCMV infection appears to negatively impact upon the host’s existing memory T cell compartment.

In order to confirm that this effect was not specific to our MCMV-Smith strain viral stocks, we repeated the experiment with MCMV stocks derived from BACS construct and purified in a different laboratory (a kind gift from Luka Cicin-Sain [29]). Similar responses were observed even at a 10-fold lower inoculum, whereby the pre-existing D8V response in Ad-lacZ immune mice dramatically decreased within 1-week post-MCMV infection (S5 Fig).

### Erosion of the existing inflating memory population occurs early after MCMV infection

**Fig 4:**
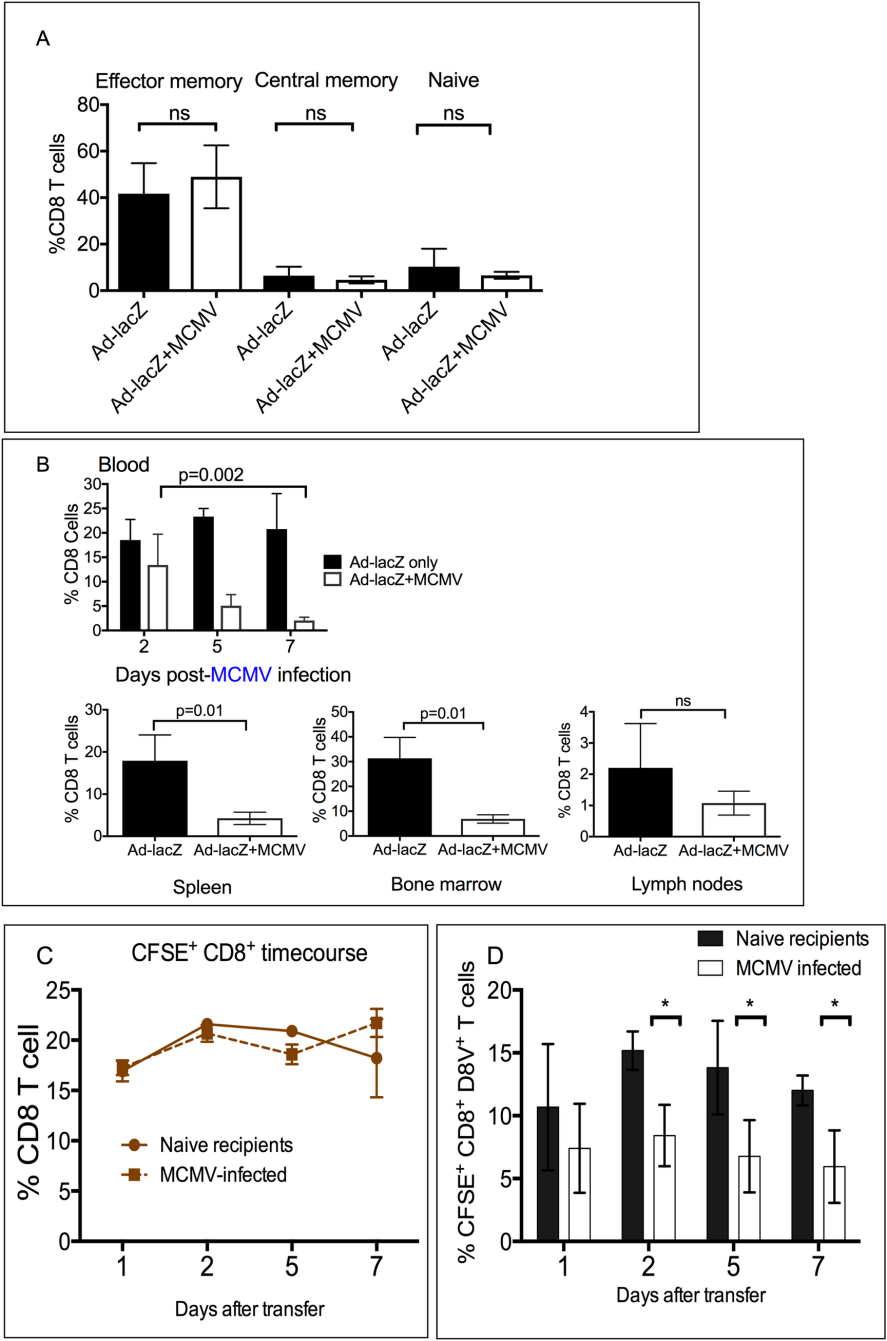
Effect of acute MCMV infection on pre-existing CD8^+^ D8V^+^ memory cells. (A) Mice were first immunized with Ad-lacZ, then infected >50 days later with MCMV. Data are combined from two independent experiments (N = 7–9 mice per group). Peripheral lymphocytes were sampled at 21 days after MCMV infection and stained *ex vivo* with CD8, CD44 and CD62L. The proportions of effector memory (CD44^+^ CD62L low), central memory (CD44^+^CD62L^+^) and naïve (CD44low CD62L^+^) was determined. (B) Levels of D8V-specific tetramer population was measured between days 2–7 after infection with MCMV in the blood (N = 4–5 from two independent experiments) and at day 4 in the spleen, bone marrow, lymph nodes and liver. (C) *In vivo* CTL assay. Splenocytes from mice immunized with Ad-lacZ 45 days previously were isolated and labelled with CFSE then equal numbers of CFSE-labelled splenocytes were adoptively transferred into mice infected with MCMV at day 1 post-infection or a group of uninfected controls. The levels of transferred D8V^+^ effecter memory cells was followed in the blood over time by *ex vivo* tetramer staining (N = 6 per group from two independent experiments). To ensure equal numbers of splenocytes were transferred, the percentage of CFSE^+^CD8^+^ cells in naïve and MCMV-infected recipients were compared at the indicated time points. (D) The levels of the inflating effector memory D8V tetramer^+^ population was measured in naïve mice and a group acutely infected with MCMV at the indicated time points. p values were measured by Mann-Whitney test. *p<0.05.

Persistent CMV and MCMV infection alters the proportion of naïve, central and effector memory subsets. Here, we observed that Ad-lacZ immunization also alters the proportion of effector, central and naïve subsets in blood in a similar manner. Compared to naïve animals, Ad-lacZ immunization caused a large expansion of the effector memory compartment, and also increased the central memory pool resulting in a decrease in the percentage of the naïve compartment (Fig 4A and S6A Fig). In mice already immunized with Ad-lacZ, a second infection with MCMV does not cause any further alterations to the proportions of these memory compartments (Fig 4A).

In initial time-course experiments of Ad-lacZ-MCMV infected mice, attrition of the D8V-specific population was seen even at the earliest time point of day 7 post-MCMV infection. We therefore measured earlier time-points to determine how soon after MCMV infection the depletion of the D8V subset occurred. As shown in Fig 4B the D8V-specific effector memory population in a number of compartments is reduced by 4 days post-MCMV infection indicating that the early phase of MCMV infection is responsible for the reduction observed. We therefore examined whether acute MCMV infection produces an environment in which effector CD8 T cells are more readily killed. Splenocytes were isolated from mice immunized 45 days previously with Ad-lacZ, labeled with CFSE and then equal numbers were transferred into naïve controls or MCMV-infected mice at 24 hours post-infection. The size of the transferred CFSE^+^D8V tetramer^+^ population in the blood was followed in both groups. As shown in Fig 4C, in both groups the percentage of transferred CFSE^+^ CD8 T cells remained stable throughout the experiment. However, in MCMV-infected animals, there is a reduction of the transferred D8V tetramer^+^ cells, which occurs soon after infection in blood and other tissues (Fig 4D and S6B Fig), thus indicating that acute MCMV infection may cause depletion of memory T cells.

### Analysis of death pathways following MCMV infection

**Fig 5:**
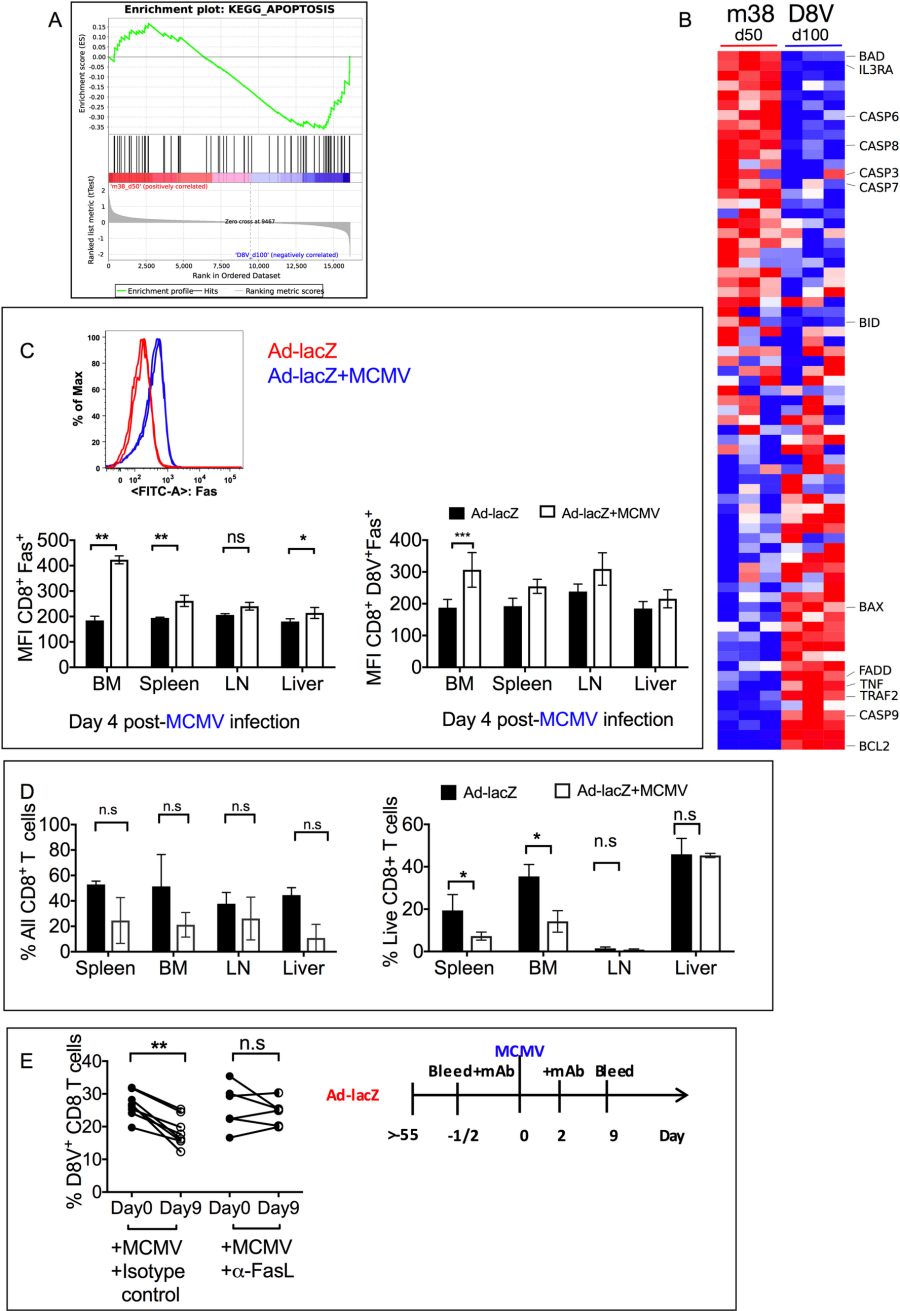
Analysis of death pathways after MCMV infection. (A) Microarray profiling of MCMV specific M38 CD8^+^ T cells and Ad-lacZ specific D8V CD8^+^ T cells was used for GSEA analysis for the KEGG apoptosis pathway comparing M38 inflationary cells at day 50 post infection to D8V inflationary cells at day 100 post infection. (B) Heatmap of the KEGG apoptosis pathway geneset from Fig 5A. Columns represent M38 or D8V samples and rows different upregulated (red) or downregulated (blue) genes. (C) A representative histogram of the levels of Fas on CD8 T cells isolated from bone marrow of Ad-lacZ only or Ad-lacZ-immune mice infected 4 days previously with MCMV. Levels of Fas on the surface of CD8 T cells (lower left) and D8V^+^CD8 T cells (lower right) isolated from the indicated organs at 4 days post-MCMV infection. (N = 4–5 per group from two independent experiments). (D) Single cell suspensions prepared from organs of mice at 4 days post-MCMV infection were cultured in vitro in complete RPMI for 2 days. The samples were gated on total CD8 T cells and the percentage of live CD8 T cells (left) and the percentage of D8V^+^ tetramer positive cells (right) within the live CD8 T cell population in each organ is shown. The data are from 4–5 mice per group from two independent experiments). (E) Mice were immunized with Ad-lacZ, then left for more than 55 days. One group of mice were injected i.v. with 100μg anti-FasL or isotype control antibody per mouse either one day before or on the same day as infection with 1x10^6^pfu MCMV. A second antibody treatment was performed 3 days post-MCMV. Blood was collected 9 days later and the levels of D8V^+^ CD8^+^T cells measured. Data from two independent experiments are shown. Each datapoint represents an individual mouse. p values determined by T-tests **p<0.005.

To investigate why D8V^+^ inflating cells may be susceptible to death in the face of acute MCMV, the expression profile between MCMV-specific M38 and Adenoviral-specific D8V^+^ inflating cells at the steady state, day 100 and day 50 post-infection respectively [22](GEO: GSE73314) were analysed. Apoptotic pathways play a prominent role in controlling the size of the memory CD8 T cell compartment; as such we performed GSEA (Gene Set Enrichment Analysis) on the two groups to determine whether there was a difference in the expression levels of genes in apoptosis pathways. As shown in Fig 5A, a subset of apoptotic genes were enriched in the D8V^+^ population compared to M38 inflating cells. While pro-survival genes including Bcl-2 were enriched in D8V^+^ cells (Fig 5B), notable apoptotic mediators including FADD, TRAF2, Bax and caspase 9 were also preferentially upregulated in the D8V^+^ population, at day 100, in the steady state. FADD and TRAF2 are components of the extrinsic pro-apoptotic pathway, and at day 4 post-MCMV infection, we detected upregulation of the death receptor Fas on the surface of CD8 T cells from various compartments, most prominently on bone marrow and splenic CD8 T cells (Fig 5C) and also on D8V specific cells in the bone marrow and lymph nodes. Consistent with this these lymphocytes were less able to survive in unsupplemented culture media (Fig 5D). To confirm the role of death pathways in mediating attrition of the D8V^+^ population, Ad-lacZ-immune mice were treated with blocking anti-FasL antibody at the time of MCMV infection. As shown in Fig 5E, blocking Fas-FasL interaction preserved the D8V^+^ population during acute MCMV infection.

### MCMV-mediated inflating memory attrition occurs in an IL-18 independent manner

**Fig 6:**
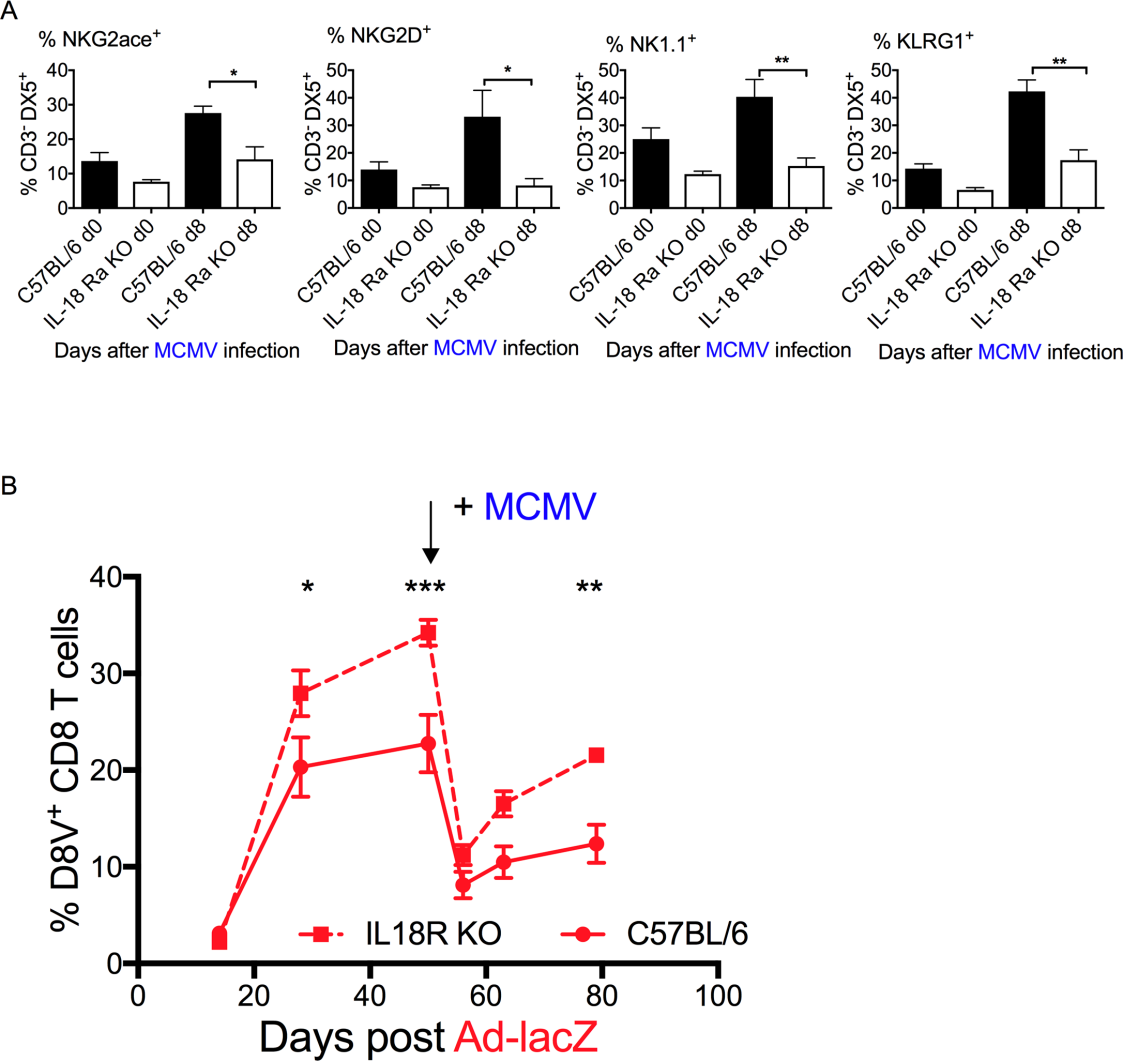
Effect of MCMV infection on pre-existing D8V responses in Ad-lacZ- immune IL-18KO mice. (A) NK cell activity in IL-18 mice upon MCMV infection. The levels of activation markers NKG2ace, NKG2D, NK1.1 and KLRG1 (left to right) were measured by *ex vivo* staining of blood after MCMV infection (N = 8–12 mice per group from 2 independent experiments). (B) IL-18RKO mice were immunized with Ad-lacZ, then 50 days later were infected with MCMV (as indicated). The levels of pre-existing D8V^+^ CD8 T cells in the blood of IL-18R KO mice (N = 5 per group) were measured by *ex vivo* tetramer staining. p values were determined using one-way Anova. *p<0.05, **p<0.005, ***p<0.0005.

Innate activation can impair the host adaptive response to infection [1–9,30,31][10–12,32]. Mice lacking the IL-18 receptor (IL-18R KO) have impaired activation of the NK cell subset, with reduced levels of the activation markers NKG2ace, NKG2D, NK1.1 and KLRG1 (Fig 6A). Making use of IL-18R knock-out mice, we repeated the co-infection experiment (Fig 6B). IL-18R KO mice still experienced attrition of the effector memory population to a similar level as wildtype (WT) controls (67% vs. 64% reduction p = n.s) suggesting that attrition occurs via an IL-18R independent pathway.

### Acute vaccinia infection does not lead to attrition of pre-existing inflating memory

**Fig 7:**
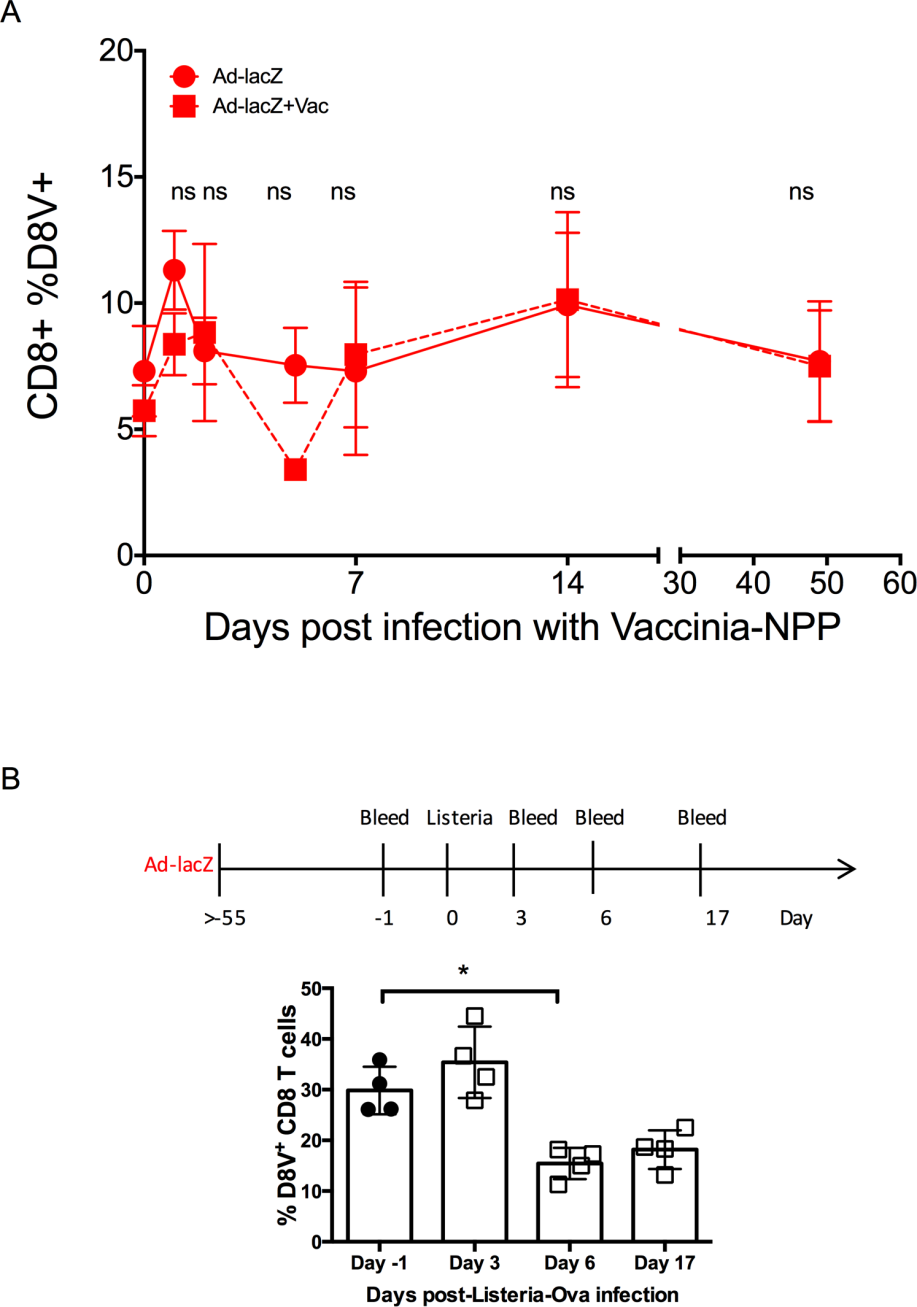
Effect of other infections on pre-existing Ad-lacZ memory responses. (A) C57BL/6 mice were first immunized with Ad-lacZ and then 50 days later were infected with vaccinia i.p. The levels of pre-existing CD8^+^D8V^+^ in the blood after vaccinia infection was measured by *ex vivo* tetramer staining (N = 3 from one of two independent experiments). (B) Mice were immunized with Ad-lacZ, then >55 days later were infected with i.v. with 1x10^3^cfu/200μl Listeria-OVA. Blood was collected at the indicated timepoints, and the levels of CD8^+^D8V^+^ cells measured by surface staining. p values were obtained using T-tests * p<0.05.

Data shown above indicated that the host is able to accommodate the presence of multiple populations of inflated memory T cells arising from sequential MCMV and Ad-lacZ infections. Therefore, the attrition of the D8V-specific population after MCMV infection cannot be explained by a limit in the size of the T cell memory compartment. As the attrition of the effector memory population occurred so early, we questioned if this was a global innate effect due to the inflammatory environment induced during a viral infection. While Ad-lacZ is an infectious virus, it does not replicate *in vivo* and therefore may not induce a similar cytokine profile to other infectious viral pathogens. We therefore repeated the same sequential experiment but this time infected Ad-lacZ memory mice with a different pathogen, vaccinia, instead of MCMV (Fig 7A). The D8V response in the blood was followed for >50 days after vaccinia infection. Here, we found that aside from a transient reduction at day 3, the percentage of D8V T cells remained largely unchanged throughout the course of the vaccinia infection. This was also observed in the populations in the liver and the lung (S7A Fig). Likewise, the size of the conventional memory response I8V was not altered by subsequent vaccinia infection (S7B Fig). Critically, we did not observe early depletion of the pre-existing memory cells or long-term reduction of the pre-existing memory population. By contrast, when Ad-lacZ memory mice were infected with the intracellular pathogen Listeria-OVA, a reduction in the size of the D8V^+^ tetramer population was also observed, albeit at a smaller magnitude compared to MCMV infection (Fig 7B). These results indicate that not all acute infections are able to impact upon pre-existing inflating memory populations in a similar manner.

Nonetheless, this would imply that during the lifespan of the host, depending on the infection history, multiple episodes of attrition of the pre-existing inflating memory pools may be experienced.

### The D8V-specific inflating memory population remaining after MCMV infection maintains an effector memory phenotype and boosting with a second dose of Ad-lacZ recovers the depleted population

**Fig 8:**
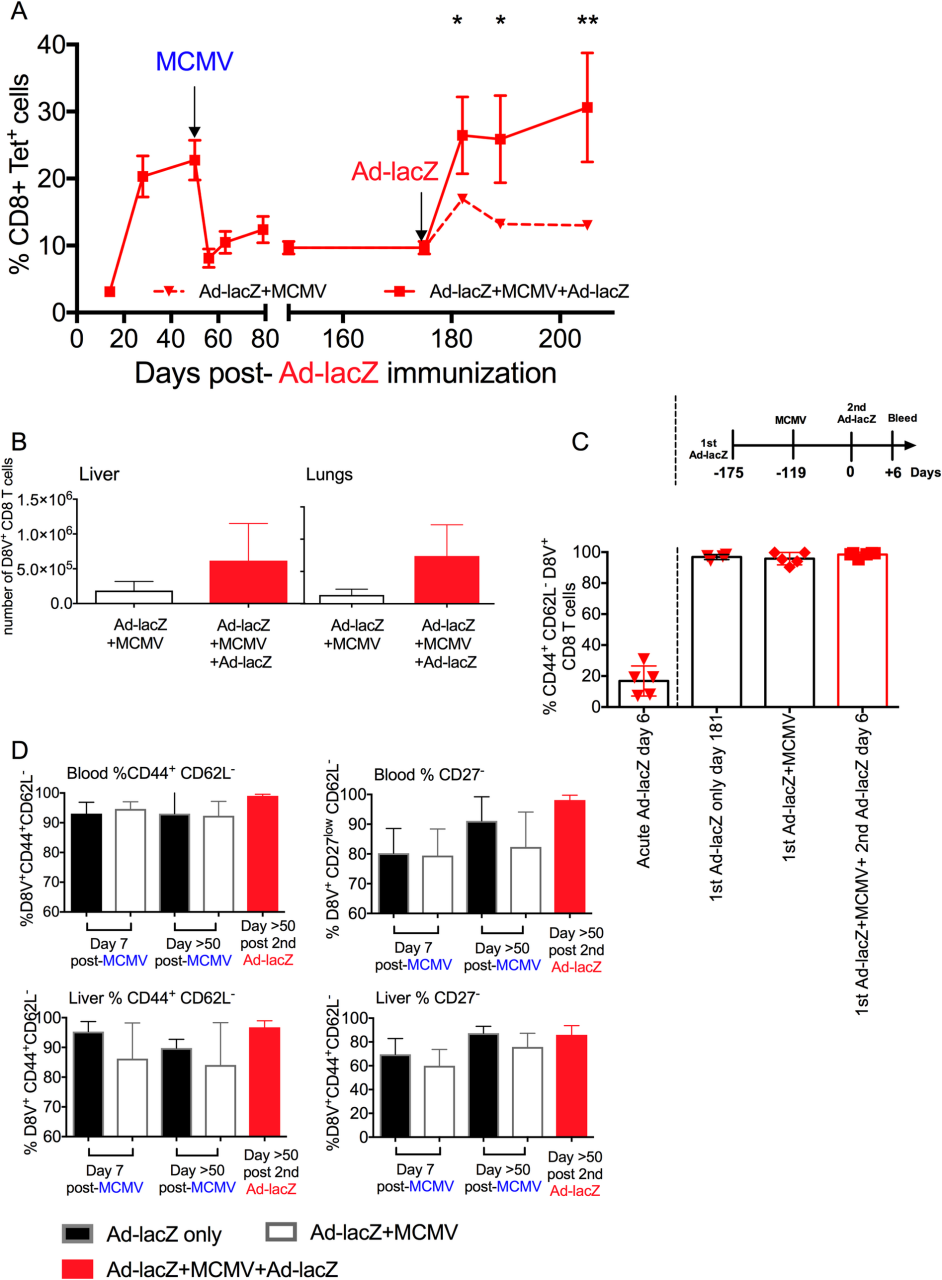
Effect of boosting with Ad-lacZ on the depleted responses. (A) C57BL/6 mice were first immunized with Ad-lacZ and then >50 days later were infected with MCMV i.v. After >50 days post- MCMV infection, the mice were boosted with a second dose of Ad-lacZ i.v. The levels of CD8^+^D8V^+^ in the blood was measured by *ex vivo* tetramer staining after primary Ad-lacZ infection. The data shown are from one of two independent experiments (N = 3). (B) The distribution of the boosted cells in non-lymphoid organs were measured at day 50 after 2^nd^ dose of Ad-LacZ. (C) The proportion of CD44^+^ CD62L^-^ expression in *ex vivo* peripheral blood 6 days after primary Ad-lacZ immunization, and 6 days after second dose of Ad-lacZ. (N = 4–6 mice from 2 independent experiments). (D) The figures show the proportion of CD44^+^CD62L^-^ (left column) and CD27^-^CD62L^-^ (right column) expression in CD8^+^D8V^+^ cells from the blood (upper row) and liver (lower row) of Ad-lacZ only, Ad-lacZ+MCMV and Ad-lacZ+MCMV boosted with Ad-lacZ groups, as measured by ex-vivo staining. The data are from two or more independent experiments (N = 4–11). p values were measured by two-way Anova followed by Sidak’s multiple comparison test. *p<0.05, **p<0.005.

It has been reported that antigen-specific boosting increased the size and persistence of effector memory cells [13–15,33]. In light of these observations we questioned whether the MCMV-depleted Ad-lacZ-induced inflating memory population might be recovered by boosting. Therefore Ad-lacZ memory mice that were subsequently infected with MCMV were later injected i.v. with a second dose of Ad-lacZ. Boosting with homologous vector successfully recovered the depleted D8V population and the population size was maintained for long periods afterwards in the blood (Fig 8A), liver and lungs (Fig 8B). To determine the level of contribution of newly primed naïve cells in the expansion, we measured the levels of T cell receptor rearrangement excision circles (TRECs) in the D8V^+^ population by quantitative PCR (qPCR). TRECs are stable, not duplicated during mitosis, and diluted out with each cellular division [34]. If the expansion following boosting was the result of increased proliferation of the existing memory pool, then the levels of TREC in the tetramer^+^ population would be lower compared to the Ad-lacZ+MCMV group. By contrast, expansion due to recruitment from the naïve pool would increase the level of TREC in the D8V^+^ memory pool. Comparing the levels of TREC by qPCR in D8V^+^ tetramer ^+^ cells 7 days after Ad-lacZ boosting with the unboosted Ad-lacZ+MCMV group indicated that TREC levels after boost was marginally higher (1.3 fold increase relative to Ad-lacZ+MCMV) than in the unboosted Ad-lacZ+MCMV (S1 Table) indicating some input from *de novo* primed cells. However naïve cell priming could not account for the full magnitude of the increase, suggesting that the expanded population could constitute of a mix of both expanded pre-existing cells and *de novo* primed cells.

To address this point further we analysed the phenotype of the boosted cells. Prior to boosting, the depleted D8V^+^ population still maintained an effector memory phenotype (Fig 8C), being mostly CD44^+^CD62L^-^ and downregulating CD27 and KLRG-1^+^ (S8A Fig) and lacking CD103 expression (S8B Fig)[16,35]. Analysis of the boosted D8V cells at the early timepoint Day 6 post-boost indicated that almost all (98.4%) of the D8V^+^ tetramer cells were still of the effector memory phenotype, akin to the D8V^+^ tetramer cells in long term Ad-lacZ immune (96.8%) and Ad-lacZ+MCMV (95.8%) mice and very different to the make up of the D8V tetramer^+^ population 6 days after primary Ad-lacZ immunization (Fig 8C), where CD44^+^ CD62L^-^ phenotype comprised the minority population (16.8%). This would again imply that the expanded cells originated largely from the substantial pre-existing pool rather than large-scale de novo priming of naïve CD8 T cells. This phenotype was stable in the recovered D8V population, still evident at 50 days post-boost (Fig 8D). Therefore, attrition of the pre-existing effector memory compartment by primary MCMV infection may not necessarily impact upon the protective ability of the depleted subsets, as they appear able to quickly expand and recover upon antigen restimulation.

## Discussion

Many new CD8 T cell immunization strategies exploit the ability to generate large numbers of long-lived antigen-specific effector memory cells [4,6–9,17–19,36]. This subset of CD8 T cell memory migrates into non-lymphoid tissues, such as the liver and lungs, and have been shown to be integral in controlling a number of clinically-relevant infections [3,20–22,37–39] in patients and animal models. How the host immune system copes with accommodating multiple large populations of effector memory cells at different points in time remains to be studied. For example, accumulation of CMV-specific responses over time is a well-described occurrence[23–25,40–42]. It has been suggested that there is finite immunological space and, over time, the large clonal expansion of CMV-specific T cells may occupy so much of it that there is little room for expansion of newly encountered antigens [26–28,43]. We therefore tested the capacity of the immune system to accommodate multiple inflationary epitopes from unrelated pathogens by using two different models that generate inflating memory populations in C57BL/6 mice.

Owing to the large sizes of these epitope specific populations, they served as useful surrogates to measure perturbations in the memory compartment during a variety of co-infection scenarios. This system may be more representative of the dynamics in real-life sequential infections as here naïve mice, without manipulation of the precursor T cell compartment, and a natural mouse pathogen are employed. This is in contrast to approaches where either mice transgenic for a particular epitope or where large numbers of epitope-specific naïve cells are first introduced and then their development measured[22,44].

Our studies indicate that two inflating responses from two different infections are able to develop at the same time. In scenarios of sequential infection, newly developing inflated cells did not impact upon the pre-existing population of effector T cells. Similarly, the presence of large existing MCMV-specific populations of inflated cells did not limit the size of the new developing Ad-lacZ inflating population. Thus the host is able to support multiple populations of epitope-specific T cells which may develop at different periods of time without any cost to the size of the existing or the newly generated effector memory population. In the case of conventional/central memory cells, a pre-existing MCMV infection may result in lower numbers of memory T cells forming during the early stages of other subsequent infections but does not appear to affect the population size at the later time-points. These findings are comparable to results in human studies of CMV, where primary CMV was found to induce expansion of the memory T cell compartment in children but did not affect their responses to vaccination [24,28,45]. Likewise, CMV reactivation in transplant patients resulted in an increase in the size of the effector and effector memory CD8 T cell memory compartment but this did not impact upon the development of the regenerating naïve and central memory compartment [20,46].

Nevertheless, pre-existing memory may be depleted by the acquisition of certain infections. We find that primary MCMV infection is able to cause systemic depletion of the inflating effector memory as well as central memory subsets, which do not fully recover in the long term. It has already been reported that CMV and MCMV infection alters the proportion of naïve and memory cells in the host, mainly by increasing the absolute numbers of MCMV-specific memory cells [20,40,41,45,47,48]. This was also observed in our model MCMV infection, where the proportion of the naïve subset was very much reduced after high-dose MCMV and also after persistent Ad-lacZ immunization, which also induces large numbers of inflating memory cells. It is interesting to note that the absolute number of cells increased in the organs after MCMV infection, possibly a result of using naïve specific-pathogen-free mice. However apart from diminishing the naive subset, MCMV infection also reduced the absolute numbers of pre-existing Ad-lacZ-specific effector and central memory cells in the liver and lung[29,49]. Viral infections, including herpesviruses have been reported to induce lymphopenia leading non-specific attrition of the memory response [22,50,51]. Our data indicates that acute MCMV infection induces rapid loss of effector memory population and this was observed in all the compartments surveyed, except the lymph nodes. Importantly, a concomitant increase in D8V^+^ cells was not observed in the surveyed organs, including the lymph nodes, strongly arguing against altered trafficking of these cells. Although *ex vivo* analysis of D8V^+^ cells early after MCMV infection did not detect any large apoptotic populations in the blood or tissues, bystander attrition of memory CD8 T cells after acute viral infection has been described previously in humans [52]and mice [53]. Gene expression analysis comparing genes expressed in D8V^+^ cells versus naïve CD8 T cells and also MCMV-specific inflating M38 cells indicated that genes in the apoptotic pathway were more enriched in this population, and in line with this, we observed an increase in Fas expression on CD8 T cells from Ad-lacZ immune mice upon MCMV infection. Furthermore, a greater proportion of these Fas-upregulated CD8 T cells died in culture compared to their Ad-lacZ counterparts. Furthermore, involvement of this pathway was confirmed by *in vivo* treatment with blocking anti-FasL monoclonal antibody at the time of MCMV infection, which prevented the attrition of the pre-existing D8V^+^ population after MCMV infection. As MCMV has been reported to poorly infect CD8 T cells, [54] we hypothesize that this may be an indirect effect of viral infection. Responses to simultaneous MCMV and Ad-lacZ infection indicate that MCMV does not impact on actively developing memory responses.

While this study has not able to pin down the full pathway leading to death of these cells, our results indicate that attrition is not dependent on IL-18. IL-18 is an early innate cytokine which is a key component of the inflammasome pathway and is also responsible for stimulating IFN-γ production [55,56]. In agreement with others [32] we found that NK activation during acute MCMV infection is impaired in IL-18 receptor deficient mice, leading to increased levels of viral DNA in the salivary glands of these animals (S9 Fig). Rydyznski et al [57] have reported that NK cells activated during acute viral infection inhibits the generation of long-lived virus-specific memory T cells by killing CD4 and Tfh cells shortly after infection. The increased size of the D8V-specific population after Ad-lacZ immunization may be a reflection of impairment of this process, as depleting NK cells during the acute infection increased the magnitude and quality of the memory response [30,57]. However, attrition of the pre-existing inflating population after MCMV infection was also observed in IL-18RKO mice. Further experiments directly targeting NK cell populations could explore whether NK cells have a non-redundant role in this process.

How this depletion is maintained in the long term is still unclear. After 100 days post-MCMV, the D8V^+^ pool was able to recover to pre-depletion levels when injected with another dose of Ad-lacZ virus, indicating that in the long term, the reduced pool of D8V cells which survived the MCMV-mediated attrition were not impaired and still retained the capacity to recognize and proliferate in response to antigen. Also, when followed out to 100 days post-MCMV infection (approximately 25% of a mouse’s lifespan), slow recovery of D8V^+^ CD8 T cells was observed in the liver and lung. More D8V^+^ cells accumulated in the lungs and this may be due to to the higher levels of beta-galactosidase expression in the lung compared to liver, as shown by Bolinger et al [20] leading to larger expansion of the D8V-specific population owing to increased antigen levels. Together, this would suggest that the limiting factor preventing recovery of this specific population is the availability of antigen presented by antigen presenting cells. Inflationary cells are associated with ongoing antigen presentation and recruitment and studies suggest that many of the inflationary cells are T cells that are accessible to the blood [23,54]. Maintenance of a sizeable population of effector T cells may therefore depend on contact of these cells with relevant APCs in the lymph node or other sites. During an acute MCMV infection, activated CD8 T cells and other cells in the vicinity of the activated T cells will upregulate FasL which will kill not only the D8V^+^ inflating cells but may impact on APCs with which they interact [58]. The data suggest that a pool of APCs are maintained at some level as the memory population is stabilized at a relatively high frequency. The difference observed between MCMV and Adenovirus-induced inflation in this study may reflect the ability of MCMV to replenish the pool of APCs, a situation which does not occur with the non-replicative vector. MCMV specific populations show remarkable resilience following *in vivo* depletion, likely through viral reactivation and re-encounter with relevant APCs.[59]

Attrition was also observed after i.v. infection with Listeria but not after systemic vaccinia infection or Adenovector immunization, which would indicate the differential activation state induced by different infections plays a role in developing activation induced cell death. In support of this hypothesis several groups have reported that human Adenovirus serotype 5 induces lower levels of innate cytokines than other adenoviral serotypes in mice, monkeys and human cells *in vitro* [60–62].

The results of this study demonstrate that, in addition to the type of pathogen, the timing of pathogen acquisition may also be crucial in shaping the existing memory compartment. CMV is usually acquired very early in life and our data would suggest that in this scenario CMV might have a low impact in shaping the host’s immunologic memory. By contrast, acquisition of primary CMV later in life may have a larger impact on the immunological memory. Control of clinically relevant infections such as *T*. *gondii* [37,38], *Trypanosoma cruzi* [39] and malaria [3] have been reported to rely on the long-term presence of large pools of antigen-specific effector CD8 T cells. Therefore, the findings of this study may explain how the timing of acquisition of certain unrelated infections may impact upon the efficacy of naturally acquired or vaccine-derived T cell immunity. Additionally, T cell vaccination strategies have been developed using recombinant Adenoviruses or CMV to generate protective inflating response in peripheral tissues against major pathogens and some have shown success to date, with translation into human trials [4–9]. The results here would suggest that immunization with adenoviral vectors allows for the generation of robust vector-specific memory while at the same time preserving the existing memory pool.

## Materials and methods

### Study design

This was a controlled laboratory study to address the effects of different viral infections on pre-existing CD8 T cell memory. Groups of mice were infected with Ad-lacZ or MCMV or both in sequence. The levels of pre-existing and newly developing inflating CD8 T cell epitopes were measured during the acute (day 7 to 14) and chronic phase (day 21 to day >50) of the second infection either in the blood or in organs. Experiments were typically performed in duplicate or triplicate, in groups of N = 2–5 per timepoint. In these experiments we were observing large differences in the size of the inflating responses that were maintained over time, which increased the power of detection.

### Ethics statement

Mouse experiments were performed according to UK Home Office regulations (project license number PPL 30/2235 and 30/2744) and after review and approval by the local ethical review board at the University of Oxford. Mice were cared for in accordance with institutional guidelines.

### Viruses, adenoviral vector and infection

MCMV strain (Strain Smith; ATCC: VR194) was used and kindly provided by Professor U.H. Koszinoswki, Department of Virology, Max von Pettenkofer Institute. MCMV was propagated and titrated on NIH 3T3 cells (ECACC), stored at -80°C, and injected i.v. at a dose of 2x10^6^ pfu.

Recombinant adenovirus expressed the β-gal protein under the control of the human CMV promoter (Ad-lacZ [63]). Ad-LacZ was propagated on HER-911 cells and purified with the Vivapure AdenoPack 20 (Sartorius; Stedim Biotech). Virus titer was determined in a cytopathic effect assay [63]. Ad-LacZ was stored at -80°C in PBS and injected i.v. at a dose of 2x10^9^pfus. Vaccinia virus (VVWR, ATCC: VR1354) was injected i.v. at 2x10^6^ pfu. Listeria-OVA was injected i.v. at a dose of 1x10^3^cfu.

### Mice

C57BL/6 mice (Harlan) and IL-18R transgenic knock-out mice were obtained from Dr Kevin Maloy (Oxford), kept under conventional conditions in individually ventilated cages, and fed with normal chow diet. Mice aged 6–8 weeks old were infected with Ad-lacZ or MCMV and then with the second virus or Listeria-OVA at the indicated time points.

### Treatment with anti-FasL monoclonal antibody

Mice were immunized with 1x10^9^pfu Ad-lacZ,.then >55 days later mice were injected i.v or i.p with 100μg/100μl per mouse of LEAF purified anti-FasL (clone Kay-10, Biolegend) or IgG2b isotype control (clone MG2b-57, Biolegend). At the time of treatment or 1 day later, mice were infected with 1x10^6^pfu MCMV i.v. Antibody treatment was repeated 2 days post-MCMV infection.

### Blood sampling and organ preparations

Peripheral blood lymphocytes (PBL) were sampled by tail vein puncture and collected in tubes containing FACS buffer (PBS, 2% BSA and EDTA).

Perfused livers were passed through a cell strainer (BD), and lymphocytes were purified through a Percoll (GE Healthcare) gradient centrifugation. Lungs were minced with razor blades and incubated in PBS containing 60 U/ml DNase (AppliChem) and 170 U/ml collagenase II (Life Technologies) at 37°C for 45 min. Cell aggregates were dispersed by passing the digest through a cell strainer (BD). The single cell suspension was spun down and then red blood cells were lysed with RBC buffer. The lymphocytes were stained for flow analysis.

In some experiments, the cell suspensions were cultured in 10% RPMI (Sigma) supplemented with L-Glutamine, sodium pyruvate, non-essential amino acids and HEPES buffer (all from Sigma) in a 37°C, 5% C02 incubator prior to flow analysis.

### Flow cytometry

Single-cell suspensions were generated from the indicated organs, and cells were incubated with the indicated mAb at 4°C for 20 min. For PBL samples, erythrocytes were lysed with FACS Lysing Solution (BD Pharmingen). Cells were analyzed by flow cytometry using a BD LSR II flow cytometer and FlowJo (Treestar), gated on viable leukocytes using the live/dead fixable near-IR dead cell stain kit from Invitrogen. MHC class I monomers complexed with M38 (H-2Kb), M45 (H-2Db), βgal D8V (H-2Kb) I8V (H-2Kb), m164 (H-2Dd) and pp89 (H-2Ld) were produced at the NIH Tetramer Core Facility (Atlanta, Georgia, USA) and tetramerized by addition of streptavidin-PE (BD Bioscience) or streptavidin-APC (Invitrogen). Aliquots of 100μl of whole blood were stained using 50μl of a solution containing tetrameric class I peptide complexes at 37°C for 20 min followed by staining with mAbs. Antibodies were obtained from eBioscience, BD Bioscience, BioLegend, Abcam, R&D Systems, and Jackson ImmunoResearch Laboratories.

### In vivo CTL assay

Splenocytes were prepared from 4 mice infected with Ad-lacZ for 47 days. Spleens were passed through a cell sieve to generate single-cell suspension. Suspension was subjected to RBC lysis, spun down then the lymphocytes counted. The cells resuspended in PBS. These were labeled with 10μl CFSE at 10M, washed, counted then resuspended in DPBS. The cell suspension was passed through a 40μl cell sieve just before *in vivo* transfer by i.v. injection. Recipient mice were either infected with MCMV for 22 hours or naïve controls. Each mouse received 2x10^7^ CFSE-labelled splenocytes in 200μl volume.

### Gene set enrichment analysis

GSEA was performed using the Broad Institute java desktop application (http://software.broadinstitute.org/gsea/) [64,65]. Analysis used the KEGG apoptosis gene set from the Molecular Signature Database v6.0 [64]sourced from http://www.genome.jp/kegg/pathway.html. Microarray data used for GSEA was from sorted MCMV specific T cells at day 50 post infection and Ad-lacZ specific T cells at day 100 post infection and can be found at GEO: GSE73314).

### Measuring TREC DNA in sorted tetramer positive cells by qPCR

Peripheral blood was collected from groups of mice (N = 3 pooled per group). RBC lysis was performed and then lymphocytes stained with tetramer-PE. Positive selection using anti-PE beads (Miltenyi) was performed and purity confirmed by FACS analysis. Purity of CD8^+^ Tetramer^+^ cells were between 87–98%. Genomic DNA was extracted from sorted cells using the Miniprep Blood DNA kit (Qiagen) and quantitated. Taqman qPCR (ThermoFisher) measuring TREC DNA was performed using the protocol and B6-specific primer and probe sequences described by Broers et al [66]. To compensate for variations in input DNA, the constant region of the TCRA (Cα) gene was used as endogenous reference gene. Relative quantitation was performed using the 2^-ΔΔCT^ equation.

### Statistical analysis

Statistical data analysis was conducted using GraphPad Prism6 software (San Diego, CA, USA). The Student’s T-test, ANOVA and the Mann-Whitney tests were employed where indicated. A p value of <0.05 was considered statistically significant.

## Supporting information

S1 Fig Inflating CD8 T cell responses in F1 (C57BL/6 x Balb) hybrids after MCMV infection. (A) Mice were infected with MCMV i.v., then levels of the MCMV-specific epitopes in the blood were measured by tetramer staining. (B) 100 days post-MCMV infection, intracellular cytokine secretion assay was performed on splenocytes to measure the levels of epitope-specific CD8 T cells by IFN-gamma secretion. (Data are from two independent experiments). p values were measured by Mann-Whitney ests. *p<0.05
(TIFF)

S2 Fig Sequential infection of MCMV followed by Ad-lacZ. (A) Schematic of the sequential infection starting with MCMV and followed by Ad-lacZ. (B) Representative FACS plots showing the M38 and M45 populations after Ad-lacZ immunization. Numbers of the pre-existing M38-specific inflating T cells in (C) the lungs and central memory M45-specific T cells in (D) the lungs, (E) blood and (F) liver was measured at the indicated timepoints post-infection with Ad-lacZ by *ex vivo* tetramer staining. (G) Representative FACS plots of the new developing inflating memory (D8V) and central memory (I8V) tetramer positive populations in the blood. The kinetics and magnitude of the new developing D8V inflating memory response in (H) the lungs and (I) central memory I8V response in the lungs, (J) blood and (K) liver was measured by *ex vivo* tetramer staining. The figures show the mean from 3–8 mice per time point obtained from 2 independent experiments. p values were measured by Mann-Whitney tests. *p<0.05
(EPS)

S3 Fig Sequential infection of Ad-lacZ followed by MCMV. (A) Schematic of the experimental design. (B) Representative FACs plots showing the pre-existing D8V inflating memory population and I8V central memory population in the blood. Timecourses of the pre-existing D8V inflating population in (C) the lungs and I8V central memory population in (D) the lungs, (E) blood and (F) liver after MCMV infection, as measured by ex vivo staining with the relevant tetramer. (G) Representative FACS plot showing the sizes of the newly developed MCMV inflating (M38) and central memory (M45) CD8 T cells in the blood over time. The kinetics of developing M38-specific inflating memory, (H) in the lungs and the developing central memory M45 response in (I) the lungs, (J) blood and (K) liver was measured by *ex vivo* tetramer staining. The figures show the mean from 3–8 mice per time point obtained from 2 independent experiments. p values were measured by Mann-Whitney tests. *p<0.05, **p<0.005
(EPS)

S4 Fig Level of the Ad-lacZ inflating epitope D8V in the peripheral blood after MCMV reinfection or infection with a lower dose of MCMV. (A) C57BL/6 mice were first immunized with 1x10^6^ pfu MCMV, then >50 days later were immunized with 2x10^9^ pfu Ad-lacZ i.v. After another >50 days later the mice were reinfected with 1x10^6^pfu MCMV i.v. *Ex vivo* tetramer staining of peripheral blood lymphocytes was employed to measure the levels of the inflating Ad-lacZ D8V population after the second infection with MCMV. (B) Levels of the Ad-lacZ inflating epitope D8V in the peripheral blood after infection with a low dose of MCMV. C57BL/6 mice were first immunized with 2x10^9^ pfu Ad-lacZ i.v. and then >50 days later with infected with 100pfu MCMV i.v. The levels of the Ad-lacZ inflating epitope D8V was measured at the indicated timepoints after MCMV infection by *ex vivo* tetramer staining. Data shown are from one of two independent experiments (N = 3 per group). T-tests were used to determine statistical significance.
(EPS)

S5 Fig Levels of D8V in the blood of Ad-lacZ immune mice after infection with 10^5^ pfu MCMV from a different laboratory. Groups of C57BL/6 mice were first immunized with 2x10^9^ pfu i.v. After >50 days, the mice were infected with 1x10^5^ pfu MCMV from a different lab. The levels of the pre-existing inflating epitope D8V in the peripheral blood was measured by *ex vivo* tetramer staining after MCMV infection. Data shown are combined from two independent experiments (Ad only, N = 4; Ad+MCMV, N = 6). p values were measured by one-way ANOVA followed by Dunn’s multiple comparison. * p<0.05.
(EPS)

S6 Fig The percentage of naïve, central and effector memory populations after individual or coinfection with Ad-lacZ and MCMV. (A) Representative FACS plots showing the gates used to determine the percentages of naïve, central and effector memory population in peripheral blood after single Ad-lacZ immunization, single MCMV infection or Ad-lacZ immunization followed by *ex vivo* staining with the CD8 and the memory markers CD44 and CD62L. (B) The numbers of transferred CFSE^+^CD8^+^D8V^+^ cells in the indicated tissues in naïve or MCMV-infected mice at 7 (N = 6 per group from two experiments) or 21 days post-transfer (N = 4 per group). T-tests were used to determine statistical significance. *p<0.05
(EPS)

S7 Fig The percentage of pre-existing inflating memory population in livers and lungs after vaccinia infection. Groups of C57BL/6 mice first infected with Ad-lacZ, then >50 days later were infected with 2x10^6^ pfu vaccinia i.v. and left for another 50 days. Organs were removed at day 50 post vaccinia infection and the percentages of Ad-lacZ (A) inflating D8V epitope and (B) central memory I8V epitope cells in the organs were measured by ex vivo tetramer staining. The data are from 3 mice per group.
(EPS)

S8 Fig The percentage of KLRG-1^+^ CD8^+^ D8V^+^ in blood after Ad-lacZ and Ad-lacZ+MCMV. **(A)** Levels of KLRG-1 expression in the CD8^+^ D8V^+^ population in the blood of Ad-lacZ and Ad-lacZ+MCMV infected animals >50 days after MCMV infection measured by *ex vivo* staining from two independent experiments (N = 4–6). (B) Representative flow staining and levels of CD103 in liver CD8^+^D8V^+^ lymphocytes from Ad-lacZ and Ad-lacZ+MCMV infected mice (N = 4).
(EPS)

S9 Fig The titre of MCMV in salivary glands of C57BL/6 and IL-18RaKO mice. Groups of C56BL/6 or IL-18RaKO mice were infected with 1x10^6^ pfu MCMV i.v. Mice were culled at the indicated days post-infection; half a salivary gland was snap frozen and MCMV titres measured by a viral plaque assay. The titres of individual mice in each group are shown, expressed as pfu/gm tissue. The dotted line indicates the limit of detection for the assay.
(TIFF)

S1 Table List of strain-specific T cell epitopes measured in F1 mice. (TIFF)

S2 Table Levels of TREC in D8V^+^ CD8^+^ T cell population after boosting with Ad-lacZ. Peripheral blood was collected from mice injected i.v 100 days previously with Ad-lacZ and then with MCMV at day -55 (Ad-lacZ+MCMV) and then boosted with Ad-lacZ at day (Ad-lacZ+MCMV+Ad-lacZ day 7) (N = 3 per group) and pooled. After RBC lysis the lymphocytes were treated with D8V-tetramer-PE. CD8^+^ D8V^+^ tetramer cells were positively selected with anti-PE microbeads and then genomic DNA (gDNA) was isolated. 20ng of gDNA was used as template in a qPCR specific for TRECs and also a qPCR specific TCR alpha to control for CD8 T cells. Using the relative quantitation method, TREC levels were normalized against TCR alpha. The table shows fold difference relative to normalized TREC levels in the Ad-lacZ+MCMV group.
(EPS)
