# Supplementary figures and images for: Adenoviral vaccine induction of CD8+ T cell memory inflation: Impact of co-infection and infection order
Source: PLoS Pathog. 2017 Dec 27;13(12):e1006782. doi: 10.1371/journal.ppat.1006782 (PMC5760110; doi:10.1371/journal.ppat.1006782)

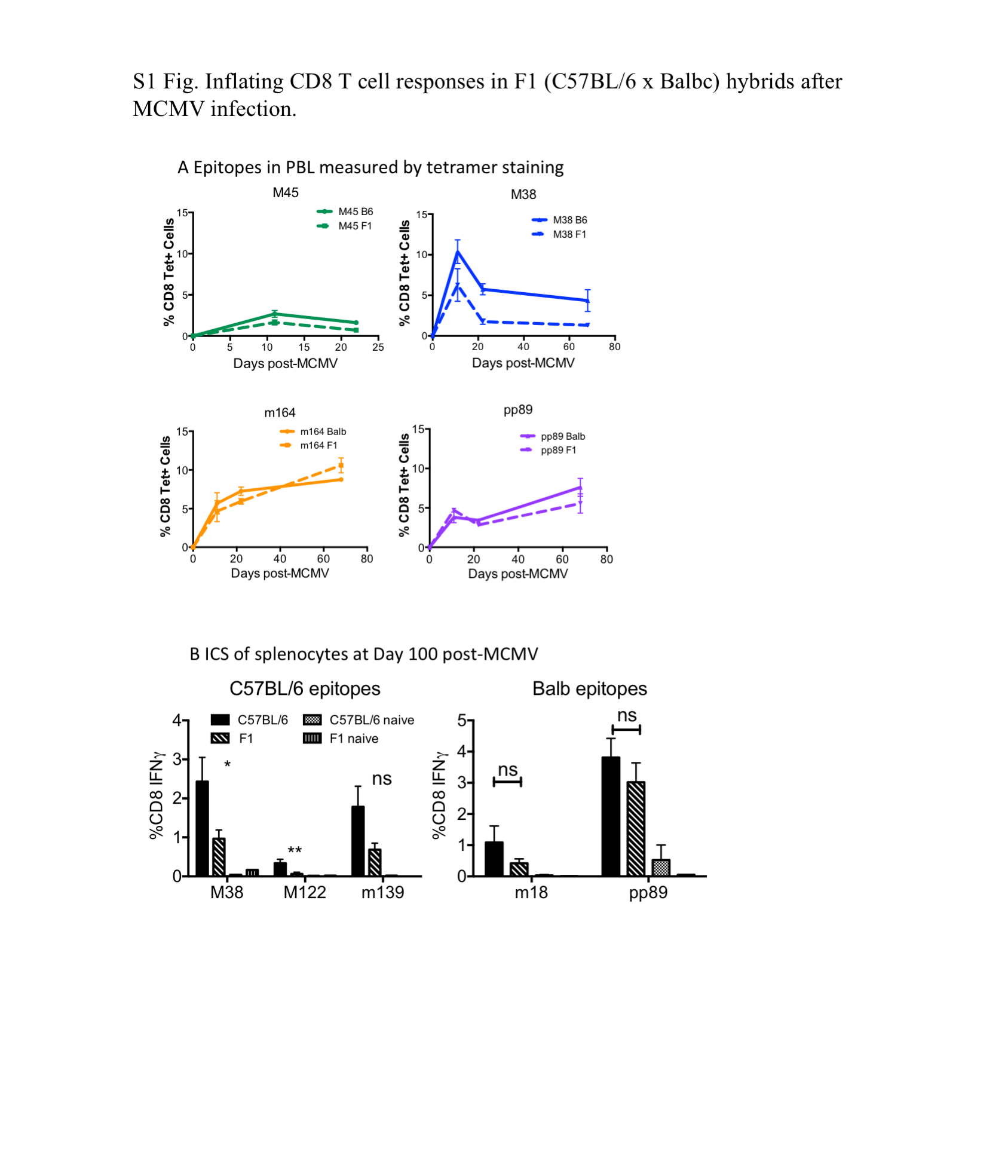

Supplement: S1 Fig — (A) Mice were infected with MCMV i.v., then levels of the MCMV-specific epitopes in the blood were measured by tetramer staining. (B) 100 days post-MCMV infection, intracellular cytokine secretion assay was performed on splenocytes to measure the levels of epitope-specific CD8 T cells by IFN-gamma secretion. (Data are from two independent experiments). p values were measured by Mann-Whitney ests. *p<0.05 (TIFF) [file ppat.1006782.s001.tiff]

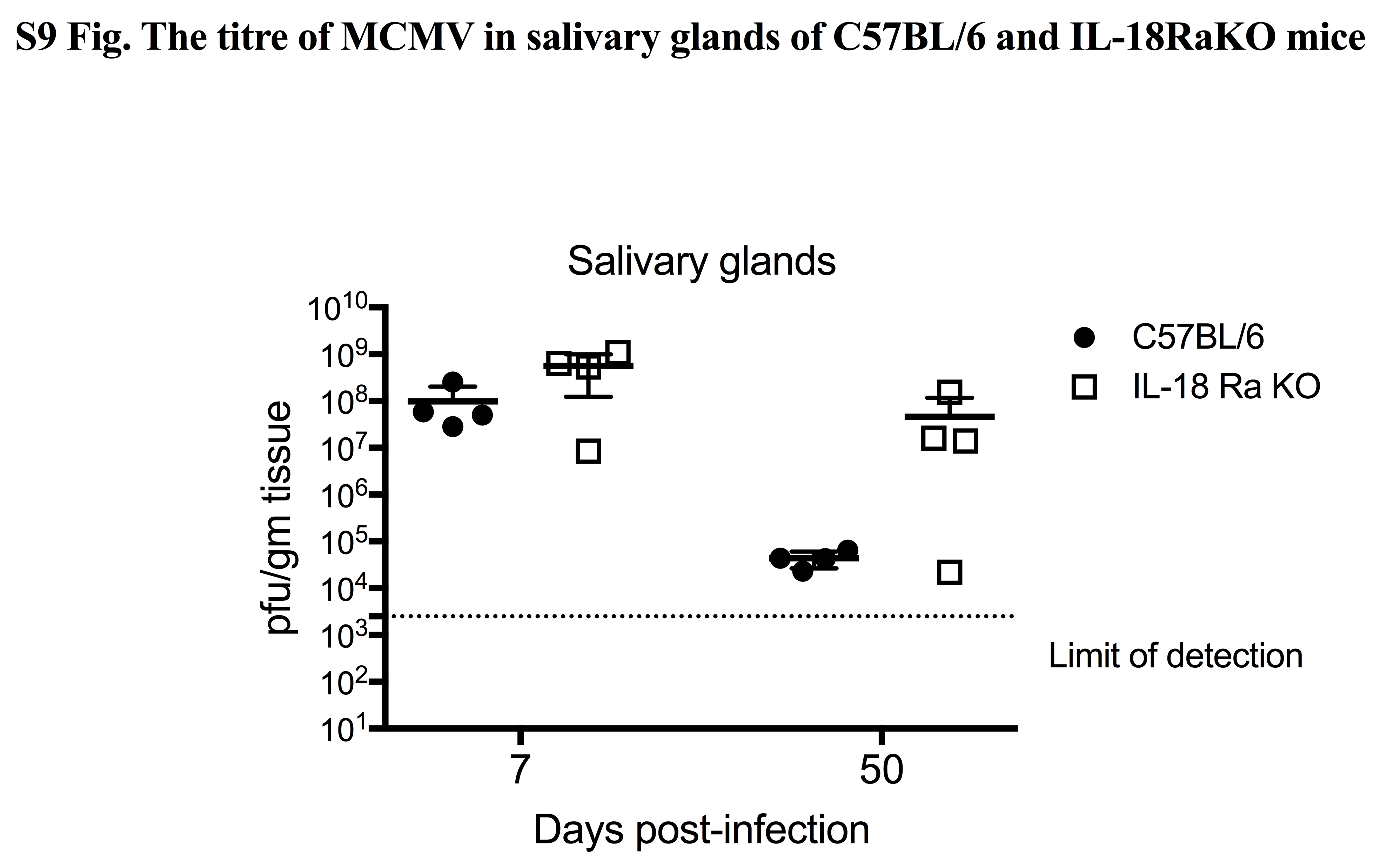

Supplement: S9 Fig — Groups of C56BL/6 or IL-18RaKO mice were infected with 1x10^6^ pfu MCMV i.v. Mice were culled at the indicated days post-infection; half a salivary gland was snap frozen and MCMV titres measured by a viral plaque assay. The titres of individual mice in each group are shown, expressed as pfu/gm tissue. The dotted line indicates the limit of detection for the assay. (TIFF) [file ppat.1006782.s009.tiff]

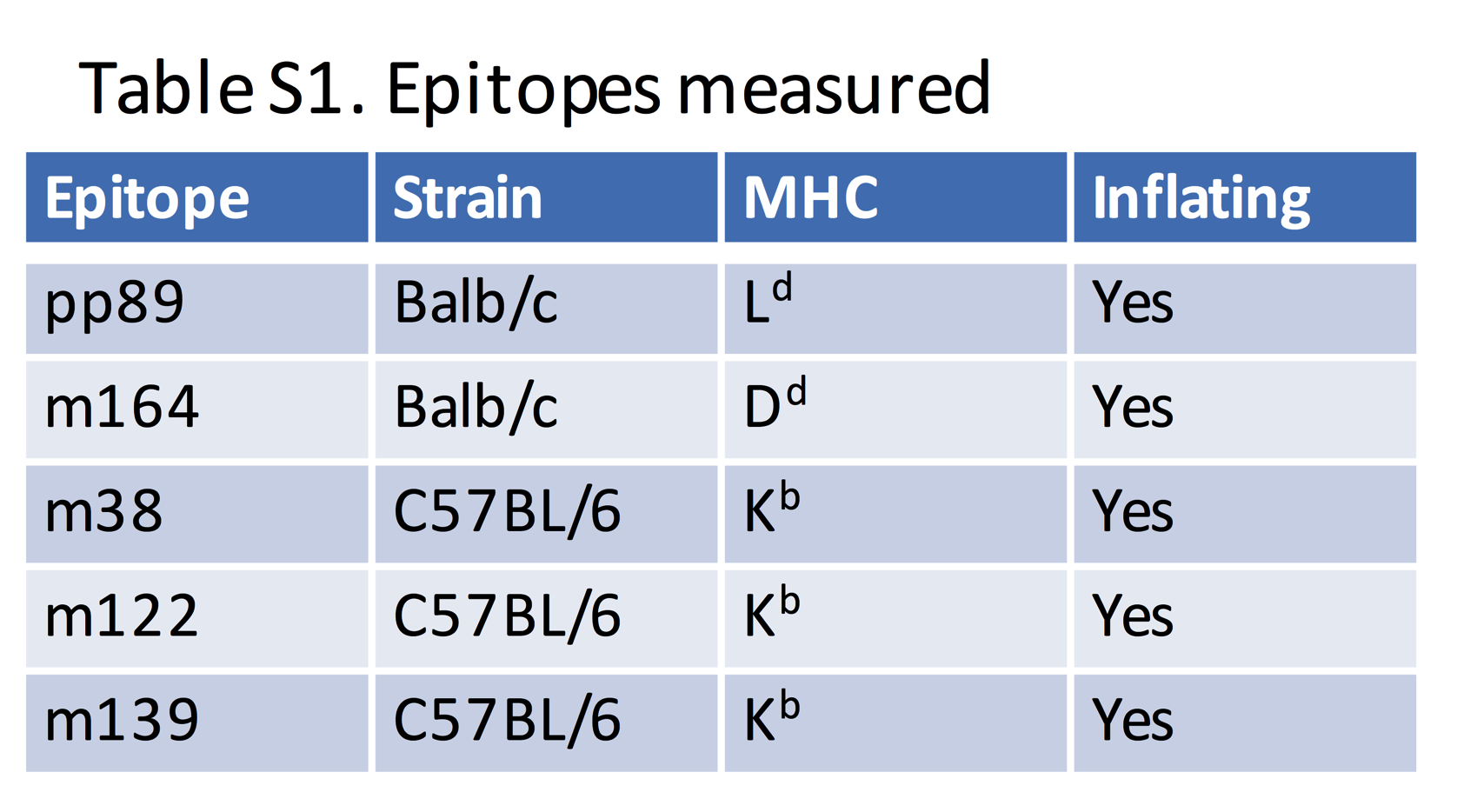

Supplement: S1 Table — (TIFF) [file ppat.1006782.s010.tiff]
